# “Paying it Forward” – Swedish Women’s Experiences of Donating Human Milk

**DOI:** 10.1177/0890334420979245

**Published:** 2020-12-04

**Authors:** Emma Olsson, Barbro Diderholm, Ylva Thernström Blomqvist

**Affiliations:** 1 6233 Department of Pediatricsm, Faculty of Medicine and Health, Örebro University, Sweden; 2 Faculty of Medicine and Health, School of Health Sciences, Örebro University, Sweden; 3 University Hospital, Neonatal Intensive Care Unit, Uppsala, Sweden; 4 8097 Department of Women’s and Children’s Health, Uppsala University, Sweden

**Keywords:** breastfeeding, breast pumping, human milk expression, milk banking

## Abstract

**Background:**

Human milk is recommended as the only nutritional source during the first 6 months of life. For preterm infants, the benefits of human milk are even more important and can alleviate the negative influences of preterm birth.

**Research aim:**

To describe how Swedish human milk donors experienced the donation process.

**Method:**

A prospective mixed methods mail survey was designed. It was sent to human milk donors (*N* = 72) at two Swedish hospitals. Quantitative data are presented with descriptive statistics and qualitative data were analyzed using qualitative content analysis.

**Results:**

The infants were between newborn and 17 weeks of age when the participants started their human milk donations, and the duration of the donation period lasted 1–24 weeks. The overall theme identified was the participants’ strong desire to help infants, often expressed as being involved in saving infants’ lives. Many participants experienced difficulties getting the information needed to become human milk donors; for others, expressing milk required both time and energy that they could otherwise spend with their own newborn infants.

**Conclusion:**

Donating human milk can be experienced as a demanding and strenuous task. Therefore, it is important that women who donate human milk receive the practical help from health care staff that they feel they need. Furthermore, information and knowledge about the possibility of donating human milk, and how important human milk is for preterm and/or sick infants, are important in order to increase the number of women willing to donate human milk.

## Background

Human milk is the ideal food for newborn infants worldwide and is recommended as the only nutritional source during infants’ first 6 months of life (Victora et al., 2016; World Health Organization, 2018). Human milk contains specific nutrients and various immunological protective factors (Palmeira & Carneiro-Sampaio, 2016). For preterm infants, the health benefits of human milk are even more important, including reducing the risk for necrotizing enterocolitis, late-onset sepsis, and retinopathy of prematurity as well as improved cognitive outcomes (Taylor, 2019). Human milk consumption can reduce the time period for which the infant needs parenteral nutrition, since infants can process greater volumes of human milk than formula (Dempsey & Miletin, 2019). Human milk also reduces the risk of bronchopulmonary dysplasia in preterm infants in comparison with a diet of formula and/or bovine milk-based fortifier (Taylor, 2019).

Recommendations have stated that all preterm infants weighing under 1500 g should receive pasteurized donor human milk (DHM), if the mother’s own milk (MOM) is unavailable (Parker et al., 2019). When MOM is not accessible, DHM is preferred over formula. DHM is human milk donated by a woman who is not the biological mother of the infant receiving the milk. MOM is almost always preferred to DHM because the pasteurization process degrades many of the elements of human milk that are protective for infants (Meier et al., 2017). Having access to DHM can affect the consumption of MOM in neonatal intensive care units (NICUs), so that infants will receive a smaller proportion of MOM and instead receive DHM. Education of mothers and NICU staff about the differences between MOM and DHM are central to increasing lactation success, and thereby increasing the consumption of MOM for infants in need of NICU care (Parker et al., 2019).

Human milk banks are institutions, established worldwide, that collect, process, and distribute donated human milk. To offer preterm infants DHM, the milk banks are dependent on available donors willing to donate human milk. Doshmangir et al. (2019) reported in a recent systematic review that the most important facilitators for women donating milk were excess of human milk, altruism, and a willingness to help other infants, while the most important barriers to donating milk were religious and cultural factors. Candelaria et al. (2018) found that human milk donors felt proud that they provided hope for infants and families. Support from nursing staff was essential, and the donating women felt that the NICU staff were important facilitators and also motivated the women throughout the donation process. Furthermore, the women described how the donation process made them feel confident, which motivated them to “give back” and maintain their donations of human milk. All experienced the donation as a positive, valuable, and nurturing experience.

Access to DHM is important in NICUs. If MOM cannot be given for some reason, DHM is the next best option and is always preferable to infant formula (Israel-Ballard et al., 2019). In Sweden, shortages of DHM sometimes occur, possibly due to its short shelf-life which makes it difficult to store large amounts. It therefore requires a stable inflow of available donors. In Sweden, parents are entitled to a total of 480 days of paid parental leave and most infants are directly breastfed at the breast. Most woman expressing human milk are usually those whose infants are cared for at an NICU. Swedish women are only allowed to donate human milk during the first 3 months after delivery. The aim of this study was to describe how Swedish human milk donors experience the donation process. By building knowledge in this area, health professionals can increase their support for women who want to donate human milk, hopefully increasing the number of human milk donors.

## Methods

### Design

A prospective, cross-sectional, mixed methods, mail survey using qualitative questionnaires with quantitative elements was designed in order to increase knowledge about Swedish women’s experiences of donating human milk. Our study was approved by the Research Ethics Committee in Sweden (Dnr 2019-02447).

**Key Messages**

- In Sweden, there are sometimes shortages of donated human milk. We therefore investigated how Swedish human milk donors experienced the donation process.
- Swedish human milk donors have a strong desire to help infants, often expressed as being involved in saving infants’ lives.
- The negative experiences of being a human milk donor included the effort and time spent making the donations.
- Emotional and practical support from healthcare providers to women donating human milk is important.

### Setting

In Sweden, the target group for DHM use is primarily preterm and sick newborn infants cared for in NICUs during their first days after birth and later during the newborn period (Milknet, 2016). At Swedish NICUs, MOM is given to infants fresh or after freezing and defrosting; there is no tradition of heat-treating MOM. Neither sampling for bacteria nor hygiene checks in general are conducted for MOM, unless there is a suspicion of infection. Almost all DHM is pasteurized before use, and all donors are screened with blood tests for HIV-1, 2, HTLV-I, II, and Hepatitis B and C. Nearly all DHM handled in Sweden occurs in NICUs, which usually have their own local milk bank. DHM availability is limited during some periods in Sweden, despite the 28 human milk banks in the country (Milknet, 2016). Furthermore, in Sweden, full-term, healthy newborn infants cared for in the postpartum care unit or at home very rarely receive DHM. In Sweden, 95% of all children are breastfed to some extent at 1 week of age, 84% at 2 months and 27% at 1 year (Socialstyrelsen [The National Board of Health and Welfare], 2019). Of the children cared for at an NICU, 80% are breastfed to some extent by discharge (Swedish Neonatal Quality Register, 2020). Mothers in Sweden who are expressing human milk for medical reasons, for example, having a preterm or sick infant in an NICU, or who are human milk donors can usually borrow a breast pump for free from an NICU. Varying financial compensation of SEK100–250 SEK (US$12–30) per liter of donated human milk is paid to the donor (tax free).

### Sample

During the first week in September 2019, all 105 human milk donors during the years 2017 and 2018 at two referral university hospital milk banks, located in the middle of Sweden, were sent a letter with a question about study participation with the questionnaire. These potential participants were identified through the human milk bank registers, and they donated an average of 8.68 L ([0.36–77.52]; *SD* 11.85) of human milk each. The hospitals were selected for reasons of convenience as the researchers worked at these hospitals. The response rate was 69% (*N* = 72). The sample size was considered adequate, as it allowed the researchers to reach data saturation in the analyses.

### Measurement

All three researchers developed the mail questionnaire with questions based on their clinical experience and literature (Candelaria et al., 2018; Doshmangir et al., 2019) establishing face validity. To address reliability, it was pilot tested with 17 Swedish human milk donors in 2015 to ensure that the questions were understood as intended. After this, only minor spelling corrections were made to the questionnaire.

The questionnaire (see Supplemental Material) consisted of seventeen questions; eight were regarding the participants’ background (demographic) information, and nine were open-ended questions about the participants’ experiences of being human milk donors. If the participants required more space to answer the questions, they could write on the back of the questionnaire.

### Data Collection

This was a paper survey and a letter detailing the aim of the study and an invitation to participate was sent by post to all prospective participants together with the questionnaire and a pre-paid reply envelope in July 2019. All participants were informed that their participation was voluntary and that they, by answering the questionnaire, agreed to participate in the study. The invitation letter contained study information, the voluntary nature of participation, that the questionnaire was anonymous (un-coded), how to contact the researchers if any questions arose, and stated that completion of the questionnaire was considered as consent. All prospective participants were sent a reminder, together with a pre-paid reply envelope, after 4 weeks.

The three researchers are experienced NICU staff, two registered nurses and one a neonatologist, working at the two study sites (NICUs). Regarding aspects (e.g., power differentials between participants and researchers and the researcher’s relationship with the participants; Dodgson, 2019), an anonymous questionnaire was assumed to be preferable as the participants would hopefully be honest in their answers.

### Data Analysis

The demographic information about the participants was analyzed using statistics. The answers to the open-ended questions were analyzed using qualitative content analysis (Lindgren et al., 2020). In this analytical process the authors read the responses several times to obtain an understanding of their content. The text was then sorted into meaning units, with word constellations each containing one piece of information. Meaning units were coded based on the content, and codes were clustered together to summarize the data. Codes for overlapping content sharing a commonality were grouped into subcategories and categories. One theme emerged—according to Lindgren et al. (2020) a theme is the unifying red thread running throughout categories and brings meaning to the phenomenon studied. The authors, throughout the entire analysis process, discussed and reflected on the understanding of the data until consensus was reached.

## Results

### Characteristics of the Sample

The participants were between 24 and 41 (*M* = 32) years old and 11 (15.3%) had previously been human milk donors (Table 1). Sixty-nine percent (*n* = 50) of their infants were full-term at birth, 25 (35%) needed neonatal intensive care (Table 2), and 22 (31%) were preterm and born after a gestational age *M* = 30.4 ([25–35]; *SD* 2.95) weeks. The infants were between newborn and 17 (*M* = 3.8; *SD* 3.23) weeks of age when their mothers started the human milk donations, and the duration of the donation period lasted 1–24 weeks (*M =* 8.7; *SD* 5.94).

**Table 1:** Demographic Characteristics of the Participants (*N* = 72).

Characteristic	*n* (%)
Older children at home	43 (59.7)
Care after delivery:	
Maternity ward	52 (72.2)
At home after “early discharge”	20 (27.8)
Previous human milk donor	11 (15.3)

*Note*. Care after delivery = the participant was cared for after the birth of her child. Early discharge = the participant and infant left the hospital 6–24 hr after birth, continuing maternity care at home with home visits by a midwife and a follow-up visit at the hospital when the infant was about 72 hr old.

**Table 2:** Demographic Characteristics of the Participants’ Infants (*N* = 72).

Characteristic	*n* (%)
Full-term infant	50 (69.4)
Care after birth:	
At home after “early discharge”	19 (26.4)
Maternity ward	28 (38.9)
NICU	25 (34.7)

*Note*. Care after birth = the participant’s infant was cared for after birth. Early discharge = the participant and infant had left the hospital 6–24 hr after birth, continuing care at home with home visits by a midwife and follow-up visit at the hospital when the infant is about 72 hr old. NICU = neonatal intensive care unit.

### Participants’ Descriptions of Their Experiences

An overarching theme identified during the analytical process was the participant’s strong desire to help infants, often expressed in terms of being involved in saving infants’ lives (Table 3). Many participants experienced difficulties getting the information they needed to be human milk donors; for others, expressing human milk required both time and energy that they could otherwise spend on their own newborn infants. Although it was sometimes perceived as demanding to be human milk donors, the participants chose to continue to donate since they genuinely wanted to help other infants. Participants’ descriptions were categorized into supporting factors and challenges and are presented below with associated codes (Table 3).

**Table 3:** Theme, Definition of Theme, Categories, Definition of Categories, Subcategories, and Definition of Subcategories.

Theme	Definition	Categories	Definition	Subcategories	Definition
A strong desire to help infants	All participants who had been a human milk donator had a strong desire to help infants, often expressed in terms of being involved in saving infants’ lives.	Supporting factors	Participants donated human milk because they genuinely wanted to help other infants.	Helping others	A desire to help others, especially infants.
				Appreciation	Economic compensation or a thank you letter.
				Information	Information about human milk donation.
				Facilitating circumstances	The circumstances were favorable (e.g., the participant had a lot of human milk).
		Challenges	Despite challenges (e.g., lack of support and the process being time and energy consuming) the participants continued, mainly because of their strong desire to help infants.	A need to prioritize	Being a human milk donator was a priority.
				Lack of practical and psychological support	Lack of support from the healthcare staff.
				A demanding task	Some part of the donation process was both time consuming and stressful.

### Supporting Factors

**Helping others**. Altruism was a common reason why the participants donated human milk. They described a desire to help others, and many also had past experiences with their older children or the children of family and friends who had received DHM during neonatal care. The participants expressed a sense of “paying it forward” and said that it was important for them to be human milk donors so that other infants could receive the same benefits as their relatives or they themselves had. One participant said that she was born preterm and had received DHM herself. Helping infants in need was an important factor described by most of the participants. One wrote: “A small effort for me can be crucial for someone else.”

Some participants knew about the shortage of human milk at hospitals and wanted to contribute, and a few compared donating human milk to donating blood. One had lost her infant in the NICU but decided to donate the milk she had expressed while at the NICU and felt good about it: “Sure, I was incredibly sad over losing my baby, but I also felt comfort in that the milk I had produced went to something good.”

**Appreciation**. A few of the donors mentioned the economic compensation as a bonus for the work they put into the human milk donation process. On the other hand, other participants said that they would have donated the milk without economic compensation, as knowing they were doing something good for someone else was enough. One participant explained that she had received a letter saying that her donated milk had been used, which made her very happy.

**Information**. Most of the participants either had their own experiences of NICU care or had families and friends with NICU experience and therefore knew about the need for DHM, or they had received information about this need at child and/or maternal health centers. Several of the participants had received donated human milk as a child or had friends and acquaintances whose children needed donated human milk, so they understood the importance of human milk donation and knew about the need. Although many knew of the need for human milk, they struggled to find information on how to become human milk donors, for example, about who they should contact and where to call. The participants suggested the need for more information to be available about the possibility and need for human milk donation, as some of them would have wanted to donate human milk during previous breastfeeding periods if they had known about it.

**Facilitating circumstances**. Some participants had facilitating circumstances, for example, producing considerable excess milk, and a few (whose infants were cared for at an NICU) were already using a breast pump to meet their infants’ needs and donated the excess. “Since I was already expressing and the infant wasn’t using all the milk, it felt better to donate than to throw the milk away.” A few said that expressing human milk was a relief because their infants would not consume as much milk as they produced, and tension was building in their breasts. Mothers of preterm infants at the NICU in some cases had a supply of already expressed and frozen milk and donated this.

### Challenges

**A need to prioritize**. Being a human milk donor was experienced as a time-consuming process, and several of the participants concluded that it was challenging to “juggle” milk expression, breastfeeding, taking care of older children, and household chores. For example: “to prioritize it [milk expression] instead of other chores that need to be done when you have an infant and older children [was difficult].” Timing milk expression and breastfeeding—ensuring there was milk in the breasts when the infant wanted to breastfeed—and timing milk expression and family activities were said to be challenging. Several participants described how they breastfed a child from one breast while simultaneously expressing milk from the other to make the process more efficient. A major challenge was finding time for and addressing the practical difficulties of both breastfeeding and expressing human milk. “It could be tough sometimes—I was uncertain how much my child had eaten. He had irregular breastfeeding times and often he was hungry just as I was expressing my milk, and that created uncertainty and stress.”

**Lack of practical and psychological support**. The healthcare staff the participants met before, during, and after being human milk donors were described by some of the participants as not always friendly and accommodating, and a few of them even expressed thoughts about not donating in the future because of this. “I could have easily done it for free. But to get a thank you, a smile, or just a cheerful response when you would leave the milk would have made a big difference.” Greater appreciation from the healthcare staff of the participants’ contribution in general was desired, and a badge or similar token that could be shown to family and friends was suggested. Several described difficulties contacting the right healthcare staff responsible for the human milk donors and felt that all the responsibility for the donating process was left to them.

Before they could even become human milk donors, many of the participants struggled to get information about who to contact and how they could become human milk donors. At the maternity wards or child health centers, information was nonexistent or outdated, according to many of the women. When the participants asked the healthcare staff about this, there seemed to be a lack of knowledge about human milk donation. According to several participants, up-to-date written information about who could be human milk donors should be available at maternity wards and child healthcare centers, and the staff of these places should have knowledge of human milk donation. “It was difficult to get in contact with the person responsible for human milk donation, which led to me having to wait a couple of weeks before I could get there and take samples and download material.”

Some said that they themselves spent time tracking down information about the human milk donation process, which was demanding work when caring for a newborn infant. To become human milk donors, a mandatory blood screening for HIV and hepatitis was required, and one participant reported having to repeat the testing at every new hospital she and her infant were transferred to; coordinating this would have been helpful, she maintained.

Many described practical challenges regarding long distances to the hospital and difficulties finding parking when delivering the human milk, while simultaneously taking care of a newborn. One participant wrote: “If they could arrange it so someone could come and collect the milk—it is extremely difficult to go in [to the human milk bank] with a baby, stroller, and several coolers.”

Several said that providing help with human milk collection or the option of delivering the human milk to a nearby healthcare center instead of going to the hospital would make it easier to be a donor. One of the two hospitals included in the study had a minimum requirement of 3 L of donated milk, which some of the participants found very stressful: “It was very hot that summer and I was nervous if I really would produce the required volume.”

**A demanding task**. The strict hygiene requirements were described by many as difficult. Washing and sterilizing (by boiling) the pumping equipment was time consuming and associated with stress regarding uncertainty about whether or not the cleaning had been meticulous enough. For example:

What takes time and is cumbersome are the hygiene requirements, that everything should be washed and sterilized after each use. I think that is very frightening. All the extra time feels like a heavy burden, especially as a mother who has recently given birth.

There were also physical complaints about sore nipples and an increasing excess of human milk (and therefore pain in the breasts) because of the expression. The transition from both breastfeeding and expressing human milk to only breastfeeding caused some participants to develop mastitis. One participant said that her infant frequently had diarrhea due to her increased human milk production, as she both breastfed and expressed.

## Discussion

There seems to be room for improvement when it comes to disseminating information about the possibility of donating human milk in Sweden. Most of the participants had previous experience of their own infants or those of family and friends being in neonatal care and receiving donated milk, so they already knew about human milk donation. There is a difference in how information about human milk donation is disseminated at healthcare units in Sweden, but our results indicate that we might not be doing enough. The lack of support during the donation process could be addressed by offering a milk pickup service at home or allowing the women to leave the expressed human milk at a nearby healthcare facility. This was already the case at one of the two hospitals included in the study. Many participants said they did not donate human milk for the economic compensation, though a few mentioned that they would appreciate a token of some sort acknowledging their efforts. Perhaps these women could receive some sort of certificate or even a text message stating that their milk has been fed to a particular infant in need (like the messages blood donors receive in Sweden). This would also help spread information about milk donation to more potential donors, as the women share these messages with friends and family. Most of the human milk donors had an infant who was full term and did not need neonatal care. Those who previously had children in need of neonatal care stated that they wanted to “pay it forward,” being grateful that their own infants had received donated human milk. They claimed that healthcare staff at various points in the chain of care (i.e., maternity units and healthcare centers) lacked knowledge of the donation process. This problem needs to be recognized in Swedish healthcare, and more information about milk donation should be made available by healthcare personnel who meet with women during pregnancy, childbirth, and aftercare. Lack of information about human milk donation is well known, and some researchers have suggested education and awareness campaigns in order to create awareness about the importance of human milk donation (Kimani-Murage et al., 2019). To convince more women to donate human milk, the participants in our study suggested organizing a milk collection system, making it easier to donate, and improving knowledge and responses on the part of healthcare staff at various points in the chain of care. According to the participants, the possibility of being a human milk donor needs to be made higher profile in the community, with more information provided earlier to expectant mothers, as well as information about the current lack of human milk and the monumental importance it has for infants’ lives.

The main reason for donating human milk was to help infants in need. This is in line with the results of the systematic review by Doshmangir et al. (2019), who found that helping other infants was one of the most important reasons for being a human milk donor. A few of the participants compared donating human milk to donating blood, illustrating their understanding of the importance of providing human milk for preterm and sick infants.

Our results are similar to those of previous researchers regarding why women choose to donate human milk (Doshmangir et al., 2019; Oreg, 2019). The donors wanted to help other infants, and they had more milk than they needed, they knew women whose infants needed donated human milk, and were aware of the importance of human milk for young children. Although the participants were predominantly positive about donating human milk, there were nevertheless some negative experiences. Worth noting here is that Swedish women mostly breastfeed direct from the breast and those who express human milk are mainly those whose infants need care at an NICU. Practical impediments were often mentioned as challenging in the process of donating human milk which, for many women in this study, meant that they should both breastfeed their infant (direct from the breast) and express human milk for donation. The participants also felt that, although they wanted to donate and were active in the donation process, they received inadequate responses and help in starting to donate. In addition, an ethical consideration arose in some participant’s minds, with some feeling that they were giving away something that belonged to their own infant or that their infant might need later. We did not ask the participants in our study about their beliefs and religions but no cultural aspects of donating human milk where mentioned in the open answers.

The concept of a wet-nurse or exchanging human milk are customs that exist in several parts of the world (Cassidy et al., 2018; Kimani-Murage et al., 2019), suggesting an exchange of human milk between friends, rather than a medically controlled arrangement through a milk bank. This type of arrangement is not common procedure in the Swedish context where full-term infants either receive MOM and, when that is not available, formula. DHM is used for preterm and/or sick full-term infants at the NICU and is always provided through a milk bank.

The participants whose infants were being cared for in neonatal units had to express human milk for their own infants in any case, so they simply donated the excess milk, not experiencing the same hygiene stress as did the participants whose infants were not cared for in neonatal units. On the other hand, the mothers with infants in neonatal care experienced other types of stress, and researchers have shown that it can be challenging to express human milk for one’s own infant when the infant needs neonatal care (Bujold et al., 2018).

One of the units specified a minimum volume of 3 L in order to donate human milk, which seemed to evoke stress among some participants, and stress itself might have reduced milk production even further. Perhaps a certain volume should be indicated as preferable, but not made an absolute requirement. The participants were each able to donate 0.36–77 L of human milk. It is remarkable that they, at the same time as having newborn infants with all the associated chores, gave their time, effort, and human milk so that other women’s infants could thrive.

Clinical implications that can be derived from this study include, especially, the participant’s perceived lack of information about the possibility of donating human milk. This is something that the clinicians at the milk banks in Sweden need to acknowledge and act upon so all mothers of newborn infants are aware of the need for DHM and thus can make an informed decision to donate or not. To lighten donors’ loads, those responsible for milk banks should also strive to make the donation process as smooth as possible with pick-up services for the human milk and greater recognition of the women’s services, all with the intention of providing the preterm and sick infants with DHM while waiting for MOM.

It would be interesting to follow up this study with a qualitative interview study allowing us to gather even richer and more in-depth information about women’s experiences of donating human milk. We also chose to include women from two different hospitals but since we wanted to ensure the anonymity of the women, we have no way of knowing which unit the respective answers related to. The main difference between the two hospitals was that one hospital stipulated a minimum amount of human milk (3 L) required to be able to donate. A national intervention in Sweden to provide information and education to all healthcare personnel who meet pregnant or postpartum women could help them better support women who want to donate human milk.

### Limitations

The use of questionnaires entails certain limitations, for example, the potential for low response rates and the inability to ask follow-up questions. The questionnaire used has not been used previously or validated beyond face validity. Perhaps interviews could have given us more information and deeper knowledge and, above all, the opportunity to ask follow-up questions. This study is culturally specific to Sweden, a small Nordic country where it is still most common that women breastfeed directly from the breast and one possible reason for the relatively high response rate was that women who have chosen to donate human milk themselves are engaged in the topic.

## Conclusions

Donating human milk can be experienced as a demanding and strenuous task. Therefore, it is important that women who donate human milk receive the practical help from health care staff that they feel they need. Furthermore, information and knowledge about the possibility of donating human milk, and how important human milk is for preterm and/or sick infants are important to in order to increase the number of women willing to donate human milk.

## Supplemental Material

Supplementary Material 1 - Supplemental material for “Paying it Forward” – Swedish Women’s Experiences of Donating Human Milk Click here for additional data file. Supplemental material, Supplementary Material 1, for “Paying it Forward” – Swedish Women’s Experiences of Donating Human Milk by Emma Olsson, Barbro Diderholm and Ylva Thernström Blomqvist in Journal of Human Lactation
