# Supplementary material for: “Paying it Forward” – Swedish Women’s Experiences of Donating Human Milk
Source: J Hum Lact. 2020 Dec 4;37(1):87–94. doi: 10.1177/0890334420979245 (PMC7907995; doi:10.1177/0890334420979245)
Supplement: Supplementary Material 1 - Supplemental material for “Paying it Forward” – Swedish Women’s Experiences of Donating Human Milk [file 10.1177_0890334420979245-suppl1.docx]

**Supplemental Material**

*Questions in the questionnaire*

| Topic | Question |
| --- | --- |
| 1. Demographic information | Your age |
|  | Where were you cared for after birth? |
|  | Where was your infant cared for after birth? |
|  | Was your infant born full-term or pre-term? If preterm; in what gestation week was your infant born? |
|  | How old was your infant when you started being a human milk donor? |
|  | Do you have any previous children? |
|  | Was this the first time you donated human milk? |
|  | How long a period did you donate human milk this time? |
| 1. Experience of being a human milk donor |  |
|  | Describe how your milk was transported to the breast milk bank/NICU |
|  | Describe where/how you got the information about the possibility of donating human milk |
|  | What made you choose to donate human milk? |
|  | Describe how you felt being a human milk donor |
|  | Was there anything that felt especially good about donating human milk? |
|  | Was there anything that felt particularly hard and/or difficult donating human milk? |
|  | Would you be willing to donate human milk again? |
|  | Is there anything you can think of that might get more women willing to donate human milk? |
|  | Do you have anything to add that could be of value for this study? |
